# Virucidal activity of olanexidine gluconate against SARS-CoV-2

**DOI:** 10.1099/acmi.0.000812.v4

**Published:** 2025-01-13

**Authors:** Rika Watanabe, Takuma Yoshida, Hidemasa Nakaminami

**Affiliations:** 1 Department of Clinical Microbiology, School of Pharmacy, Tokyo University of Pharmacy and Life Sciences, 1432-1 Horinouchi, Hachioji, Tokyo 192-0392, Japan

**Keywords:** antiseptics, olanexidine gluconate, SARS-CoV-2, virucidal activity

## Abstract

Antiseptics have been used for infection control against severe acute respiratory syndrome coronavirus 2 (SARS-CoV-2). Ethanol (EtOH) was found to be effective against SARS-CoV-2, while chlorhexidine gluconate (CHG) was less effective. Therefore, virucidal activity may differ between different classes of antiseptic agents. In this study, the efficacy of antiseptics against SARS-CoV-2 was evaluated, and effective agents for infection control were identified. The following antiseptics were used in this study: 1.5% olanexidine gluconate (OLG); 80% EtOH; 1% sodium hypochlorite (NaClO); 0.2% benzalkonium chloride (BKC); 1% povidone-iodine (PVP-I); 0.5%, 1% and 1.5% CHG; and 0.5% alkyldiaminoethylglycine hydrochloride (AEG). The virucidal activity was evaluated at 0, 0.5, 1, 2 and 3 min according to EN14476. After 0.5 min of exposure, 1.5% OLG, 80% EtOH, 1% NaClO, 0.2% BKC and 1% PVP-I inactivated SARS-CoV-2 below the detection limit. The virus survived in the presence of 0.5% CHG, 1% CHG or 0.5% AEG for 3 min. The virucidal activity of 1.5% CHG was insufficient after 0.5 min of exposure. The results showed that virucidal activity against SARS-CoV-2 differs depending on the class of antiseptic agents used under clean conditions. Despite belonging to the same class of biguanide antiseptics, OLG was more effective against SARS-CoV-2 than CHG.

## Data Summary

All data associated with this work are reported within the article Aand supplementary files.

## Introduction

Severe acute respiratory syndrome coronavirus 2 (SARS-CoV-2) was isolated in Wuhan, China, in 2019 [1]. SARS-CoV-2 is thought to have spread through contact and droplet transmission. Therefore, the appropriate use of effective antiseptic agents is crucial for infection control [2,3]. Based on their antimicrobial spectra, antiseptic agents are classified as having high, medium or low virucidal activity. High-level antiseptics are used to disinfect medical devices, such as endoscopes, and medium-level antiseptics are used to disinfect the environment, skin and hands. Low-level antiseptics are used to disinfect wounds, mucous membranes and the surrounding environment. In 2015, olanexidine gluconate (OLG) was introduced in Japan as a biguanide surgical field disinfectant [4]. OLG is active against bacteria, including *Staphylococcus aureus* and *Pseudomonas aeruginosa*, and viruses, including the human coronavirus and influenza viruses [4,5]. SARS-CoV-2 has a lipid envelope that renders it susceptible to alcohol-based antiseptic agents [2]. In contrast, chlorhexidine gluconate (CHG), a biguanide disinfectant, was less effective against SARS-CoV-2 [2]. Therefore, there may be differences in virucidal activity between classes of antiseptic agents. In the present study, we evaluated the efficacy of antiseptics against SARS-CoV-2 and identified effective agents for infection control.

## Methods

The SARS-CoV-2 JPN/TY/WK-521/2020 strain (SARS-CoV-2^WK-521^) was obtained from the National Institute of Infectious Diseases (Tokyo, Japan). VeroE6/TMPRSS2 cells (JCRB1819) were purchased from the Institute of Biomedical Innovation, Health, and Nutrition (Osaka, Japan). VeroE6/TMPRSS2 cells were maintained in VP-SFM (GIBCO, Nebraska, USA) supplemented with 200 mM l-glutamine, 100 units ml^−1^ penicillin and 100 µg ml^−1^ streptomycin. The cell culture medium and viral stock did not contain serum or any organic material, ensuring that the study was conducted under controlled, clean conditions without the presence of interfering substances, such as blood, saliva or other contaminants. The following antiseptics were included in this study at clinically used concentrations: 1.5% OLG (molarity is unknown due to the lack of density information) from Otsuka Pharmaceutical Factory, Inc. (Tokushima, Japan); 80% ethanol (EtOH) (approximately 13.7 M) from FUJIFILM Wako Pure Chemical Corp. (Osaka, Japan); 1% sodium hypochlorite (NaClO) (approximately 28 mM) from FUJIFILM Wako Pure Chemical Corp.; 0.2% benzalkonium chloride (BKC) (approximately 5.38 mM) from FUJIFILM Wako Pure Chemical Corp.; 1% povidone-iodine (PVP-I) (approximately 0.453 mM) from Sigma-Aldrich Co. LLC (Missouri, USA); 0.5% (approximately 5.56 mM), 1% (approximately 11.1 mM) and 1.5% (approximately 16.7 mM) CHG from FUJIFILM Wako Pure Chemical Corp.; and 0.5% alkyldiaminoethylglycine hydrochloride (AEG) (approximately 13.2 mM) from FUJIFILM Wako Pure Chemical Corp. In this study, 0.5 and 1.0% CHG were evaluated as these are the antiseptic agents most commonly used for hand and skin disinfection. The antiviral activity of 1.5% CHG was compared with that of OLG, the same class of biguanide antiseptics.

The virucidal test was conducted under clean conditions without interfering substances according to EN14476 [6]. An aliquot of 50 µl of test virus was mixed with 450 µl of the test substances (or HEPES buffer as a negative control), and the samples were incubated for 0, 0.5, 1, 2 and 3 min at room temperature. No interfering substances were used in this study, and the test substances were diluted with distilled water. After incubation, the antiseptics were removed by gel filtration [6]. For the gel filtration method, 100 µl of the samples was diluted with 900 µl of PBS and filtered through a gel filtration column (columns filled with Sephacryl S-400; Cytiva, Tokyo, Japan). The filtrates were then serially diluted tenfold with the culture medium. The viral titres [log_10_ median tissue culture infectious dose (TCID_50_) per ml] in all antiseptics were measured using quantitative assays with six wells per dilution and three biological replicates using the Spearman–Kärber method [6]. The results represent the mean values of the log_10_ TCID_50_ per ml ±sd of three independent experiments.

## Results

**Fig. 1.**
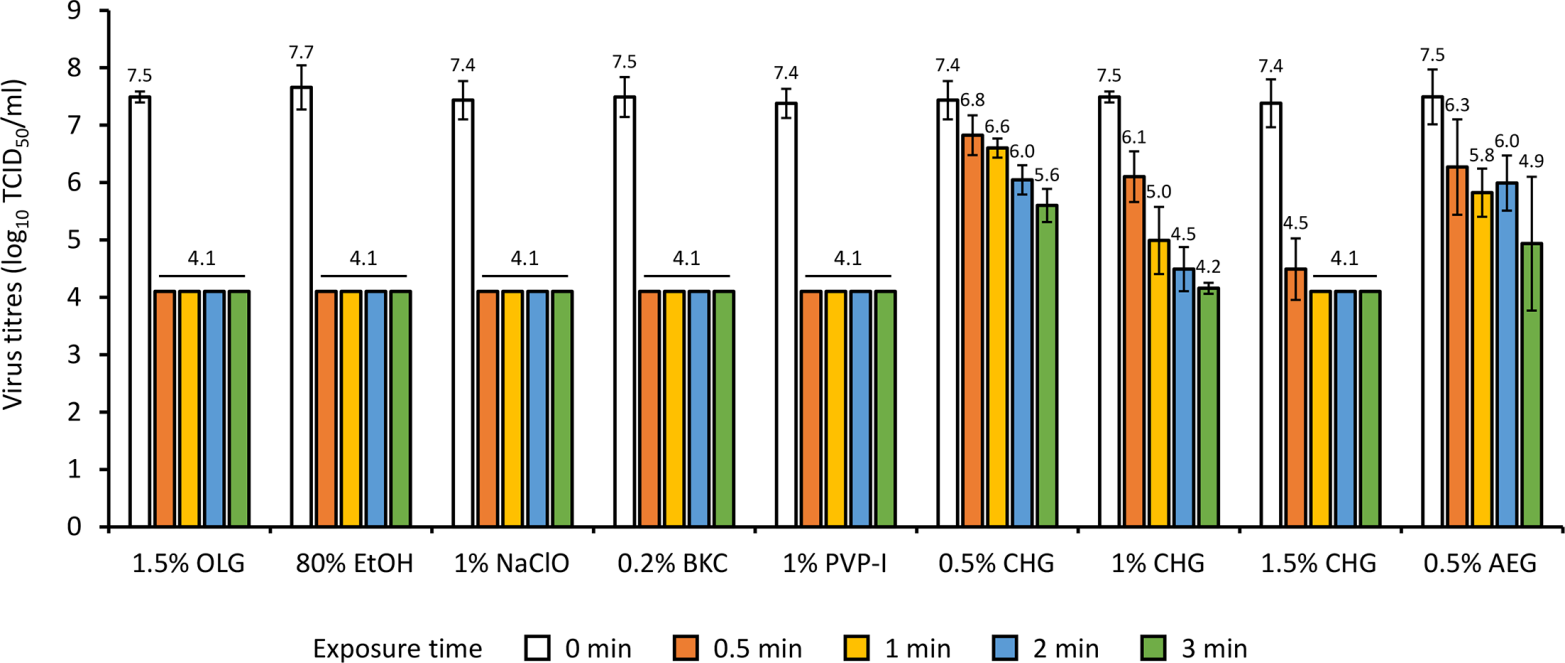
Virucidal activity of antiseptic agents against SARS-CoV-2^WK-521^. The virus titres were measured after 0–3 min of exposure to antiseptic agents. Each column and vertical bar represent the mean values of the log_10_ TCID_50_ ml^−1^±sd of three independent experimental values. OLG, olanexidine gluconate; EtOH, ethanol; NaClO, sodium hypochlorite; BKC, benzalkonium chloride; PVP-I, povidone-iodine; CHG, chlorhexidine gluconate; AEG, alkyldiaminoethylglycine hydrochloride.

Comparison of the virucidal activities of the antiseptic agents showed that 1.5% OLG, 80% EtOH, 1% NaClO, 0.2% BKC and 1% PVP-I inactivated SARS-CoV-2 below the detection limit after 0.5 min of exposure (Fig. 1). In contrast, the virus survived in the presence of 0.5% CHG, 1% CHG and 0.5% AEG after 3 min of exposure. Although the virucidal activity of 1.5% CHG was insufficient after 0.5 min of exposure, the viral activity was reduced below the detection limit after 1 min of exposure. 1.5% OLG, 80% EtOH, 1% NaClO, 0.2% BKC and 1% PVP-I demonstrated more than a 1000-fold reduction in viral titres, with an RF >3 according to EN14476. Additionally, 1 and 1.5% CHG achieved RF >3 after 3 min of exposure, while 1.5% CHG maintained RF >3 after at least 1 min of exposure.

**Fig. 2.**
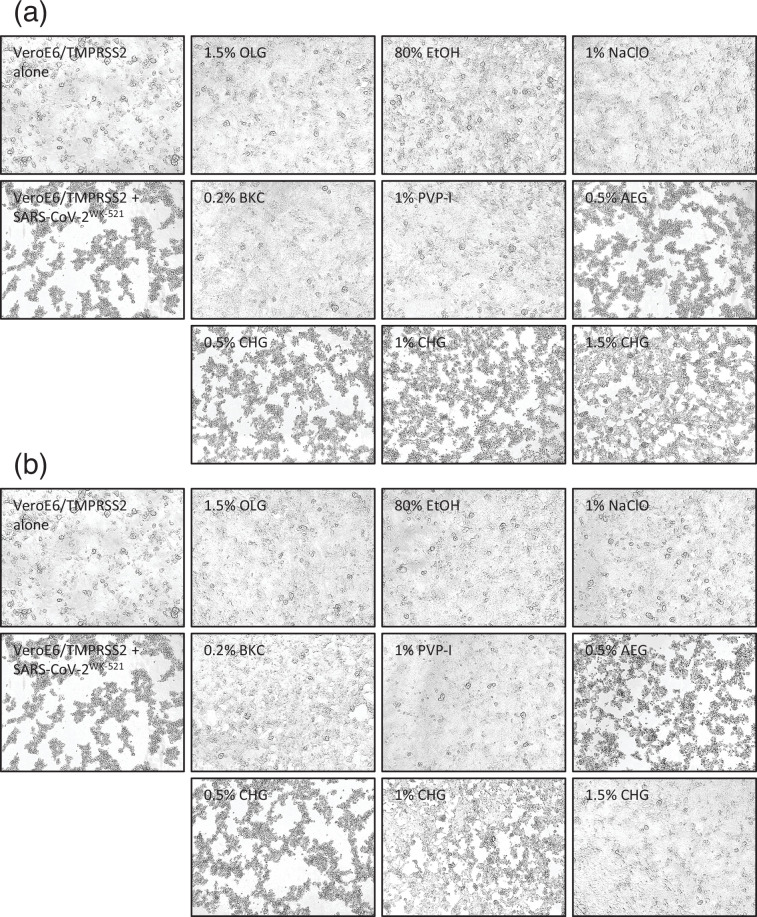
Phase contrast image of VeroE6/TMPRSS2 after 0.5 min (**a**) and 3 min (**b**) of antiseptic exposure. The images were contrast-enhanced for the purpose of publication. OLG, olanexidine gluconate; EtOH, ethanol; NaClO, sodium hypochlorite; BKC, benzalkonium chloride; PVP-I, povidone-iodine; CHG, chlorhexidine gluconate; AEG, alkyldiaminoethylglycine hydrochloride.

Cell morphology was maintained by inactivating the virus with 1.5% OLG, 80% EtOH, 1% NaClO, 0.2% BKC or 1% PVP-I for 0.5 min (Fig. 2a, b). In contrast, cytopathic effects (CPEs) were observed when the virus was exposed to 0.5% CHG, 1% CHG and 0.5% AEG for 0.5 and 3 min, owing to insufficient virucidal activity. Consistent with virucidal activity, cell morphology was maintained by 1.5% CHG with 3 min exposure to the virus, whereas 0.5 min exposure was insufficient, and CPE was observed. Viral samples treated with the antiseptic were passaged twice, and no CPE was observed in samples in which the virus was reduced below the detection limit, confirming the complete inactivation of the virus (Fig. S1, available in the online Supplementary Material).

## Discussion

OLG, EtOH, NaClO, BKC and PVP-I were found to be highly effective antiseptic agents against SARS-CoV-2. EtOH is widely used as an effective antiseptic agent against SARS-CoV-2 [7]. EtOH is most commonly used for hand hygiene because of its fast-acting properties, as well as in hospitals to disinfect medical equipment. However, it is not expected to have a long-lasting effect owing to its high volatility. NaClO, which is primarily used to disinfect medical devices, volatilizes its components in a short time. SARS-CoV-2 has been reported to survive for long periods on various surfaces, including human skin and materials [8,9]. Therefore, disinfection with EtOH or NaClO is likely to allow SARS-CoV-2 to survive if it is dried over time. In contrast, BKC and PVP-I are reported to be highly persistent and have a long-lasting effect [10]. This suggests that the effectiveness of disinfectants varies significantly depending on their persistence on object surfaces. Further studies will be needed to determine the effectiveness of disinfectants on various surfaces.

The virucidal activities of CHG and AEG were lower than those of other antiseptic agents used in this study. The low virucidal activity of CHG against SARS-CoV-2 has also been reported [2]. This is consistent with the results of a previous study showing that AEG has low virucidal activity against SARS-CoV-2. The virucidal activity of CHG increased in a concentration-dependent manner, although the virus survived even at 1.5% CHG, the maximum concentration used in this study. Despite belonging to the same class of biguanide antiseptics, OLG was more effective against SARS-CoV-2 than CHG. However, they are structurally slightly different; CHG is a divalent cationic compound, whereas OLG is a monovalent cationic compound [4]. Therefore, the difference in the charge of the compounds may affect their virucidal activity. Further studies will be needed to clarify the differences between the mechanisms of action of CHG and OLG against SARS-CoV-2 infection.

In this study, various antiseptic agents were evaluated at the maximum concentrations used in clinical settings. The results showed that most antiseptic agents inactivated SARS-CoV-2 below the detection limit. The appropriate concentrations of antiseptic agents depend on their intended use. Therefore, the evaluation of virucidal activity at low concentrations may reveal more detailed differences between antiseptic agents. Although this study was conducted under clean conditions, it is important to note that real-world disinfection scenarios often involve surfaces contaminated with organic materials, such as blood, saliva or other bodily fluids. These interfering substances can significantly affect the efficacy of antiseptic agents, potentially reducing their virucidal activity. Future studies should evaluate the performance of these antiseptics under dirty conditions to better reflect their effectiveness in practical infection control settings.

## Conclusion

Virucidal activity against SARS-CoV-2 was found to differ depending on the class of antiseptic agents used under clean conditions. Despite belonging to the same class of biguanide antiseptics, OLG was more effective against SARS-CoV-2 than CHG. Therefore, appropriate antiseptic agents should be selected for SARS-CoV-2 infection control.

## supplementary material

10.1099/acmi.0.000812.v4 Uncited Fig. S1.

10.1099/acmi.0.000812.v4 Uncited Table S1.

## Abbreviations

AEG: alkyldiaminoethylglycine hydrochloride
BKC: benzalkonium chloride
CHG: chlorhexidine gluconate
CPE: cytotoxic effects
EtOH: ethanol
NaClO: sodium hypochlorite
OLG: olanexidine gluconate
PVP-I: povidone-iodine
SARS-CoV-2: severe acute respiratory syndrome coronavirus 2
SARS-CoV-2^WK-521^: SARS-CoV-2 JPN/TY/WK-521/2020 strain
TCID_50_: median tissue culture infectious dose
