# Supplementary material for: Virucidal activity of olanexidine gluconate against SARS-CoV-2
Source: Access Microbiol. 2025 Jan 13;7(1):000812.v4. doi: 10.1099/acmi.0.000812.v4 (PMC11726771; doi:10.1099/acmi.0.000812.v4)
Supplement: Uncited Fig. S1. [file acmi-7-00812-s001.pdf]

Fig. S1

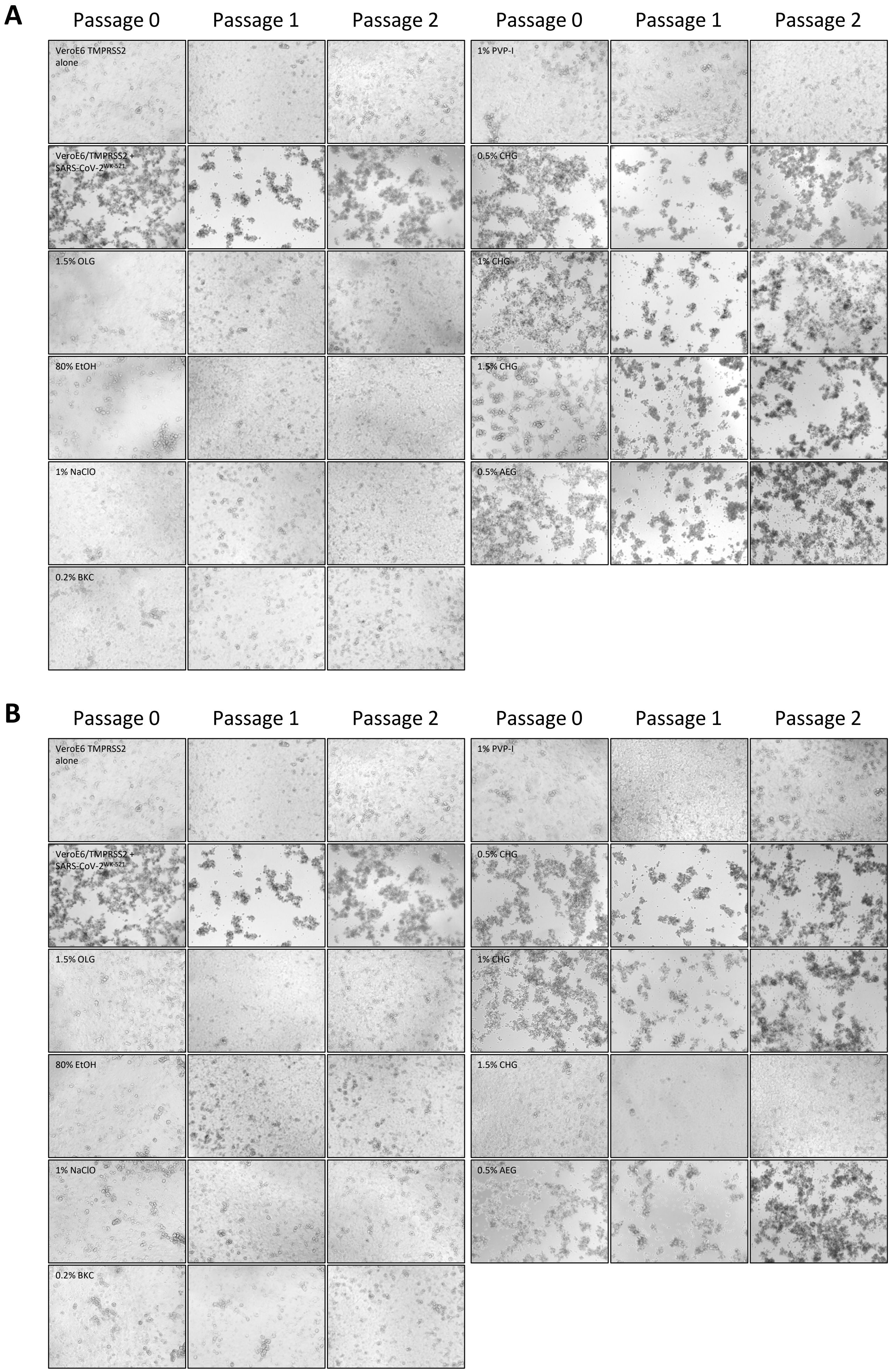
A
Passage 0
Passage 1
Passage 2
Passage 0
Passage 1
Passage 2
VeroE6 TMPRSS2 alone
VeroE6/TMPRSS2 + SARS-CoV-2WK-521
1.5% OLG
80% EtOH
1% NaClO
0.2% BKC
1% PVP-I
0.5% CHG
1% CHG
1.5% CHG
0.5% AEG
B
Passage 0
Passage 1
Passage 2
Passage 0
Passage 1
Passage 2
VeroE6 TMPRSS2 alone
VeroE6/TMPRSS2 + SARS-CoV-2WK-521
1.5% OLG
80% EtOH
1% NaClO
0.2% BKC
1% PVP-I
0.5% CHG
1% CHG
1.5% CHG
0.5% AEG

Fig. S1_Continue

C

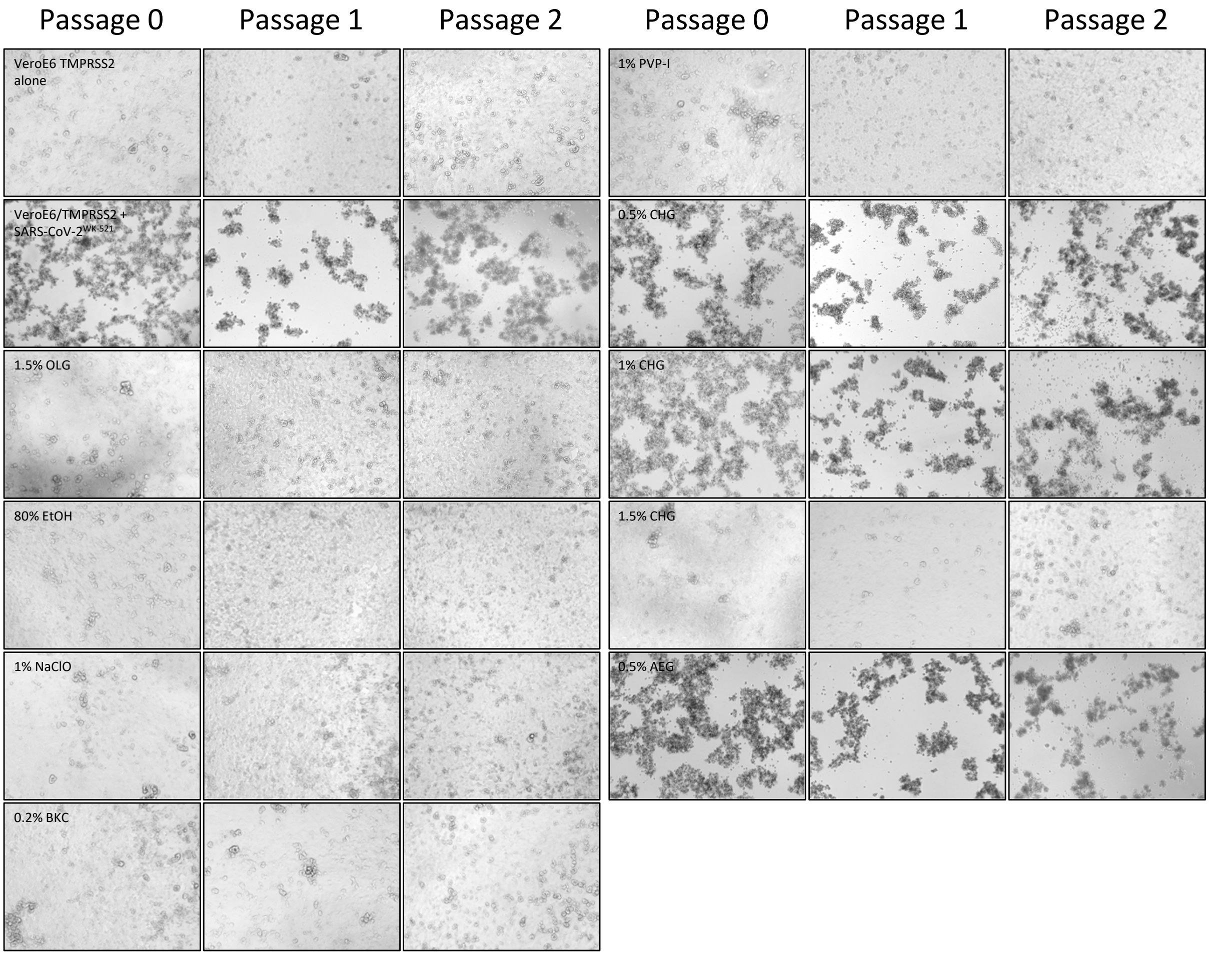

D

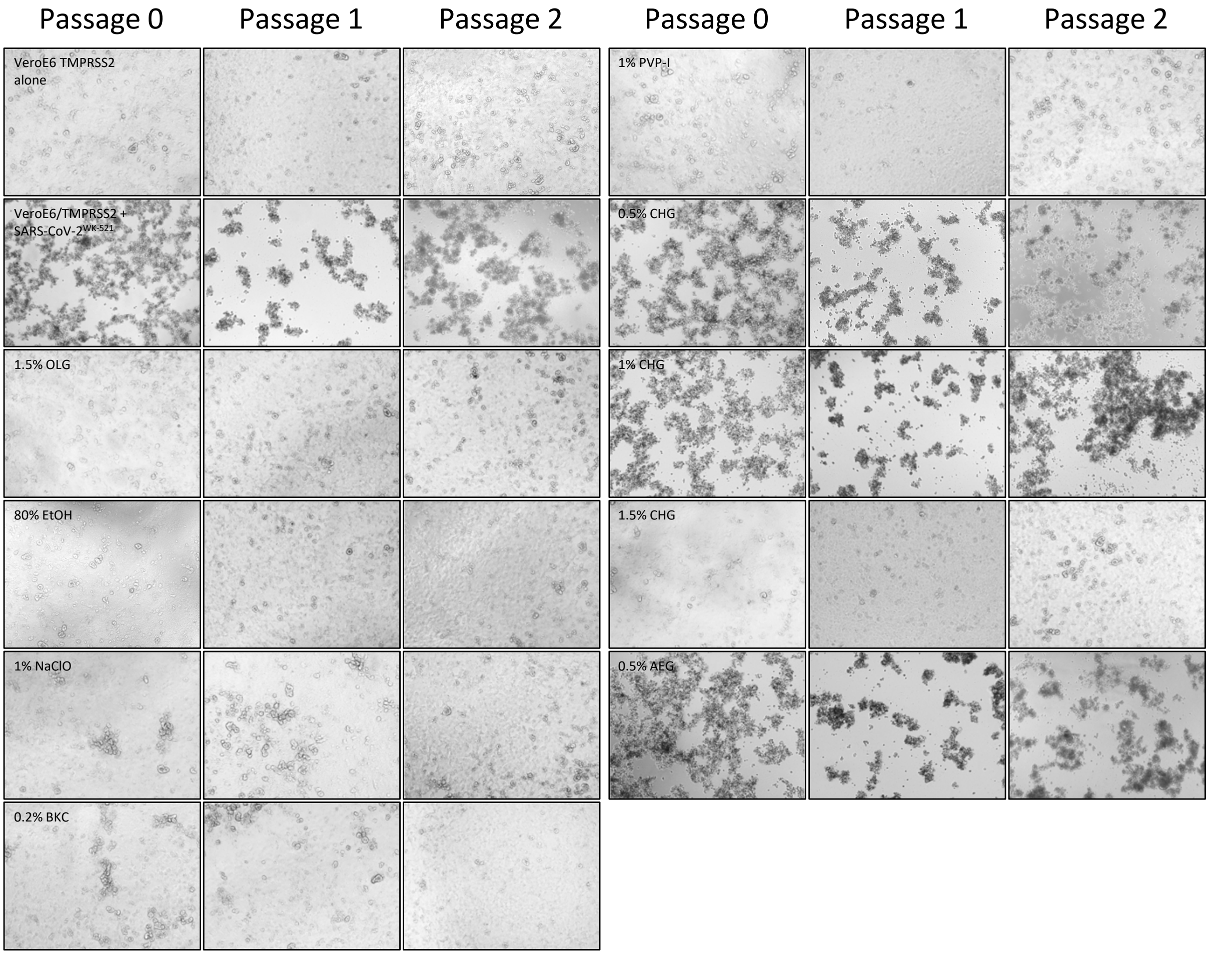

Fig. S1. Phase contrast image of VeroE6/TMPRSS2 after exposure to antiseptic agents.
Viruses exposed to antiseptic agents for 0.5 min (A), 1 min (B), 2 min (C), and 3 min (D) were passaged 0-2 times.
The images were contrast-enhanced for the purpose of publication.
OLG, olanexidine gluconate; EtOH, ethanol; NaClO, sodium hypochlorite; BKC, benzalkonium chloride;
PVP-I, povidone-iodine; CHG, chlorhexidine gluconate; AEG, alkyldiaminoethylglycine hydrochloride
